# Corneal endothelial changes in type 2 diabetes mellitus with reference to stage of diabetic retinopathy

**DOI:** 10.6026/973206300214226

**Published:** 2025-11-15

**Authors:** Pramukh Prasad U, Rashmi G

**Affiliations:** 1 Department of Ophthalmology, Sri Devaraj Urs Medical College, Tamaka, Kolar, India

**Keywords:** Type 2 diabetes mellitus, diabetic retinopathy, corneal endothelium, endothelial cell density, hexagonality, coefficient of variation, central corneal thickness, specular microscopy

## Abstract

The impact of Type 2 Diabetes Mellitus (T2DM) and diabetic retinopathy (DR) stage on corneal endothelial morphology and central corneal thickness (CCT) remains unclear. In this cross-sectional study conducted at R.L. Jalappa Hospital with 80 participants (40 diabetics and 40 controls), specular microscopy revealed that diabetic patients had significantly lower endothelial cell density, reduced hexagonality and increased coefficient of variation, indicating endothelial dysfunction. Although CCT was slightly thinner in diabetics, the difference was not statistically significant. Moreover, analysis across DR severity (No DR, NPDR and PDR) showed no significant correlation with endothelial parameters, despite a worsening trend with disease progression. Thus, we show that corneal endothelial changes in T2DM may occur independently of DR, underscoring the importance of routine corneal evaluation before intraocular procedures.

## Background:

Diabetes mellitus is a growing global health concern: India had approximately 77 million people with diabetes in 2019 and this number is projected to reach over 134 million by 2045. As a metabolic disorder it affects multiple body systems, including the eyes [1, 2]. Diabetic retinopathy (DR) remains a leading cause of vision loss globally and the principal cause of visual impairment among adults aged 25-74 years. Early microvascular changes-selective pericyte loss, capillary basement membrane thickening and microaneurysm formation-disrupt the blood-retinal barrier, increase permeability and lead to retinal oedema. Progressive capillary occlusion and arteriolar narrowing bring about retinal ischaemia, which in turn induces pathological neovascularisation, vitreous haemorrhage, fibrovascular proliferation and eventually tractional or rhegmatogenous detachment and neovascular glaucoma. Recent reviews have further characterised the molecular drivers-oxidative stress, inflammatory cytokines, VEGF upregulation, leukostasis and epigenetic modifications-that underlie these structural changes [3, 4]. Further mechanisms implicated in retinal changes secondary to diabetes include retinal microthrombosis, altered retinal blood flow, retinal neovascularization alterations, sorbital build up and advanced glycation end products [5]. Diabetes mellitus (DM) induces significant structural and functional alterations in the corneal endothelium. Even in well-controlled Type 2 DM, studies demonstrate a reduction in endothelial cell density, increased polymegethism, pleomorphism and a lower percentage of hexagonal cells, reflecting impaired endothelial stability and limited regenerative capacity. Beyond these morphometric abnormalities, the diabetic cornea is predisposed to a spectrum of clinical complications, including recurrent erosions, punctate epithelial keratopathy, persistent epithelial defects, reduced corneal sensitivity, delayed epithelial wound healing, superficial keratitis and ulceration, all of which highlight its heightened susceptibility to functional decompensation [6]. Functionally, diabetic corneas exhibit increased permeability and reduced endothelial pump efficiency, resulting in stromal hydration and higher central corneal thickness (CCT). Kim *et al.* demonstrated that patients with Type 2 Diabetes Mellitus show significantly lower endothelial cell density, increased pleomorphism and greater CCT compared to non-diabetic controls, with these changes correlating with higher HbA1c levels and longer disease duration. These findings suggest that poor glycemic control contributes to endothelial dysfunction and structural alterations of the cornea, underscoring the importance of metabolic regulation in maintaining corneal transparency [7]. Endothelial morphology is assessed using several parameters: cell density (cells/mm^2^), polymegathism (CV), pleomorphism (percentage of hexagonal cells) and mean cell area ± standard deviation (µm^2^) [8]. Specular microscopy is a non-contact imaging technique that allows *in-vivo* visualization and quantitative assessment of corneal endothelial cells, including parameters such as cell density, morphology and hexagonality, essential for evaluating corneal health and transparency [9]. Therefore, it is of interest to report corneal endothelial changes in type 2 diabetes mellitus relative to the stage of diabetic retinopathy.

## Materials and Methods:

This prospective study was designed to evaluate corneal endothelial changes in patients with Type 2 Diabetes Mellitus (T2DM) less than 60 years of age, compared to healthy controls with each group of patients consisting of 40. Participants were included based on the presence of T2DM or absence of systemic illness for the control group. Exclusion criteria encompassed prior intraocular surgery, laser treatments, periocular or intravitreal injections, history of glaucoma, uveitis, significant refractive errors (>3.0 D hyperopia or myopia, >1.0 D astigmatism), corneal disorders (e.g., keratoconus, Fuch's dystrophy, opacities, dry eye), contact lens use, chronic topical medication use and systemic diseases other than diabetes. Data were collected through a comprehensive ophthalmic evaluation that included visual acuity assessment using Snellen's chart, near vision testing, slit-lamp biomicroscopy and fundus evaluation with +90D lens and indirect ophthalmoscopy, with diabetic retinopathy graded according to the ETDRS classification. Specular microscopy was employed to assess central corneal thickness (CCT) and corneal endothelial morphology. Participants were divided into two main groups-diabetic and control. The diabetic group was further classified into three subgroups based on the stage of diabetic retinopathy: Group 1 with no DR, Group 2 with Non-Proliferative DR (NPDR) and Group 3 with Proliferative DR (PDR). All findings were documented and analyzed to evaluate the impact of diabetes and retinopathy stage on corneal endothelial health.

## Results:

In this observational study comparing diabetic patients (cases) with non-diabetic controls, there was no significant difference in age or sex distribution between the two groups, indicating demographic comparability (p > 0.05; Figure 1 - see PDF). Diabetic patients showed significantly altered metabolic and corneal endothelial parameters compared to controls. Specifically, fasting blood sugar (FBS), postprandial blood sugar (PPBS) and HbA1c levels were significantly higher in the diabetic group (p < 0.05), confirming their diabetic status. Additionally, endothelial cell density (ECD) was markedly lower, while hexagonality (HEXA) was higher and coefficient of variation (CV) was significantly lower in cases, suggesting morphological changes in the corneal endothelium due to diabetes. Central corneal thickness (CCT), however, did not differ significantly between groups (p = 0.098; Figure 2 - see PDF). When participants were grouped by retinal status (No DR, NPDR, PDR), glycemic parameters (FBS, PPBS, HbA1c) worsened progressively with DR severity (p < 0.05; Table 1 - see PDF), highlighting the link between poor glycemic control and retinal disease.

## Corneal parameters also changed significantly:

HEXA increased, while CV and ECD declined with advancing DR (p < 0.001). CCT showed no significant variation across groups (Figure 2 - see PDF).

## Discussion:

This study evaluated the impact of Type 2 Diabetes Mellitus (T2DM) on corneal endothelial morphology and its association with diabetic retinopathy (DR) severity in 80 participants (40 diabetics, 40 controls), matched for age and gender (Figure 1 - see PDF). Diabetic patients showed significant reductions in endothelial cell density (ECD), decreased hexagonality (HEXA) and increased coefficient of variation (CV), indicating endothelial dysfunction. While central corneal thickness (CCT) was slightly lower in diabetics, the difference was not significant (Figure 2 - see PDF). Stratification by DR stage (No DR, NPDR, PDR) revealed no statistically significant differences in endothelial parameters, though worsening trends were observed with advanced DR (Table 1 - see PDF). This study evaluated the impact of Type 2 Diabetes Mellitus (T2DM) on corneal endothelial morphology and its association with diabetic retinopathy (DR) severity in 80 participants (40 diabetics and 40 controls) matched for age and gender (Figure 1 - see PDF). Diabetic patients showed significant reductions in endothelial cell density (ECD), decreased hexagonality (HEXA) and increased coefficient of variation (CV), indicating endothelial dysfunction. Although central corneal thickness (CCT) was slightly lower in diabetics, the difference was not statistically significant (Figure 2 - see PDF). Stratification by DR stage (No DR, NPDR and PDR) revealed no significant differences in endothelial parameters, though a progressive worsening trend was noted with advancing retinopathy (Table 1 - see PDF). These results align with previous findings by Kim *et al.* (2021), the current findings suggest that endothelial alterations are more closely related to diabetes duration and glycemic control than to the stage of retinopathy itself [7]. Consistent with Çolak *et al.* (2021), who reported that longer diabetes duration and higher HbA1c levels were significantly associated with reduced ECD, decreased HEXA, increased CV and greater CCT in T2DM patients. Similarly, Jha *et al.* (2022) observed progressive reductions in ECD and HEXA with advancing DR severity, alongside increases in CV and CCT. In agreement, Durukan *et al.* (2020) also noted progressive ECD loss with DR severity [10, 11-12]. The results reinforce the need for preoperative corneal evaluation in diabetics, even in early or no DR stages, as endothelial changes may precede overt retinal findings. Variability in outcomes across studies highlights the complex interplay of factors such as diabetes duration, glycemic variability and systemic comorbidities. Study limitations include small sample size, cross-sectional design and lack of control for systemic confounders. Absence of advanced imaging like confocal microscopy also restricted deeper corneal assessments.

## Conclusion:

The impact of poor glycemic control on systemic and ocular health, particularly its association with diabetic retinopathy is reported. Elevated fasting, postprandial blood sugar and HbA1c levels correlated with retinal changes and altered corneal endothelial parameters such as ECD and hexagonality. Data shows the role of chronic hyperglycemia in accelerating diabetic eye disease and support the need for strict glycemic monitoring to reduce ocular complications. Despite its insights, the study's modest sample size and unaccounted confounders call for larger, longitudinal studies using advanced imaging to better understand the link between metabolic dysregulation and ocular pathology.
